# Animal-Assisted Interventions in Paediatric Hospitals: An Investigation of Italian Healthcare Personnel Attitudes

**DOI:** 10.3390/children12030352

**Published:** 2025-03-11

**Authors:** Cinzia Correale, Sofia Orlando, Marta Borgi, Simonetta Gentile, Simona Cappelletti

**Affiliations:** 1 Neurology, Epilepsy and Movement Disorders, Bambino Gesù Children’s Hospital IRCCS, Full Member of European Reference Network EpiCARE, 00165 Rome, Italy; 2 Department of Human Sciences, LUMSA University, 00193 Rome, Italy; 3 Centre for Behavioural Science and Mental Health, Istituto Superiore di Sanità, 00161 Rome, Italy; marta.borgi@iss.it

**Keywords:** animal assisted therapy, attitude of health personnel, hospitals, paediatric, COVID-19

## Abstract

Background: Evidence of the beneficial effects of animal-assisted interventions (AAI) on patients admitted to paediatric hospitals is growing. However, there is still little information about healthcare professionals’ knowledge of and attitudes towards AAI, both as a complement to medical treatments and as a tool for improving the workplace environment. The present study explores the perspectives of Italian paediatric hospital staff after the onset of the COVID-19 pandemic. Methods: An online questionnaire was developed and distributed to paediatric hospital personnel across Italy. The questionnaire addressed topics including AAI’ impact on the hospital environment, their role as a resource for patients and families, their effect on staff well-being, and the perception of the feasibility of AAI implementation in hospitals. Data were analysed descriptively and qualitatively. Results: A total of 44 respondents took part in the survey. Most respondents agreed that AAI could improve hospital environments and serve as a valuable resource for patients and families. However, results were more mixed about the effects of AAI on staff well-being and the feasibility of their implementation. Qualitative analysis identified recurring themes including the positive impact of AAI on emotions/general well-being, improved compliance and treatment outcomes, and reduced stress and distress. Concerns included organisational/logistical challenges, hygiene issues, and potential impact on staff workload. Notably, most participants felt that the COVID-19 pandemic had not affected their perception of AAI safety. Conclusions: Most respondents viewed AAI favourably and supported their implementation as a means of benefiting patients and caregivers. Concerns mainly related to organisational and logistical barriers highlight areas that require further exploration in future research.

## 1. Introduction

Over the past fifty years, animal-assisted interventions (AAI) have become increasingly popular as a non-pharmacological approach to help children cope with hospitalisation [1,2,3]. These interventions—which involve the use of animals, such as dogs, to improve patients’ physical, emotional, and social well-being—have been shown to reduce anxiety, promote relaxation, and foster social interaction [4]. Such benefits are particularly meaningful for hospitalised children, who often experience heightened stress and emotional distress during their stay [5,6].

While a growing body of research has explored the views of patients, family, and caregivers, there is limited knowledge regarding the perspectives of hospital personnel, despite their crucial role in coordinating and implementing these interventions, and their potential to benefit from AAI as well. Existing studies on healthcare personnel’s attitudes towards AAI, particularly in paediatric hospitals, are limited but have suggested generally positive views and acceptance [7,8,9,10,11,12]. Concerns regarding potential risks, such as dog bites or equipment damage, tend to decrease once the benefits of AAI become evident [7]. This is further supported by previous research, which reports no increases in hospital infections or adverse events following the introduction of therapy dogs [9,11].

However, the emergence of the COVID-19 pandemic not only affected patients’ mental health [13] but also transformed hospital environments and operational procedures [14,15]. AAI have not been exempted from these changes. Indeed, the pandemic has significantly impacted AAI delivery, leading to temporary suspensions and modifications in hospital programs [16]. These changes were particularly evident in Italy, a country that has increasingly adopted AAI [2,17] and that was one of the most affected by the COVID-19 emergency.

Given these factors, this exploratory study aims to explore the perspectives of Italian paediatric hospital staff on AAI, including their potential benefits and awareness of the logistical and safety challenges associated with their implementation following the COVID-19 pandemic. While this study represents an initial step in understanding hospital personnel’s attitudes toward AAI in the post-pandemic period, it may provide valuable insights to support its successful integration into paediatric hospital settings.

## 2. Methods

### 2.1. Sample

The study targeted a specialised population comprising paediatric hospital personnel working in Italy, including staff working in both daycare/outpatient paediatric settings and inpatient units. Participants were included if they were: (i) hospital personnel (clinical or administrative staff); (ii) working in paediatric services; and (iii) based in any region of Italy. The study employed a non-probability convenience sampling method to gather data from paediatric hospital personnel across the country.

### 2.2. Data Collection

An online questionnaire was developed and disseminated through social media, informal channels, and emails distributed throughout the Italian Paediatric Hospital Association network. The aim of this approach was to ensure widespread distribution and encourage participation from a wide range of hospital personnel. This methodological choice was guided by practical constraints, including limited funding, hospital staff availability, and pandemic-related restrictions. While in-depth interviews or focus groups could have yielded richer qualitative insights, the online survey allowed us to reach a broader and national-based sample while minimising the burden on healthcare personnel.

All participants were free to accept or decline to take part in the study. The survey was completed anonymously, and no participant identifiable data were requested. Demographic characteristics such as age and gender were not collected to protect confidentiality and encourage honest participant responses. Before starting the questionnaire, participants were provided with a section containing information on consent, including researcher contact details and assurance of anonymity. Data collection took place between August and December 2022.

### 2.3. The Online Questionnaire

The questionnaire was developed by Sofia Orlando and Cinzia Correale and was based on previous research on hospital staff members’ perceptions of AAI [12,18]. The questionnaire was subsequently reviewed by a researcher from the Italian National Institute of Health (Istituto Superiore di Sanità) to ensure clarity.

The final questionnaire consisted of 26 items organised into three sections:

A. Knowledge of AAI: This section examined participants’ knowledge of AAI through their responses to two “Yes/No” questions (regarding knowledge and experience of AAI) and two open-ended questions (answered only if the “Yes” option was selected).

B. Implementation of AAI and their benefits: This section examined participants’ opinions and perspectives on the implementation of AAI and their benefits in the hospital context through multiple-choice questions. Participants responded to a series of statements regarding AAI by selecting the option that best represented their perspective in a 5-point Likert scale from “strongly disagree” to “strongly agree”. Likert-type items were chosen to measure hospital personnel attitudes, given their widespread use in quantifying qualitative information [19]. In the multiple-choice items, Items 17, 21, and 22 were phrased negatively to minimise response bias. This section comprised 18 items investigating the following aspects: AAI and the hospital environment (4 items), AAI as a resource for patients and families (6 items), AAI and hospital staff well-being (6 items), and perceptions of AAI implementation feasibility (2 items).

C. Positive/negative aspects of AAI, acceptance and AAI implementation challenges: This section explored participants’ opinions about the positive/negative aspects of AAI and challenges to their adoption, as well as their willingness to adopt AAI (acceptance) through responses to four open-ended questions.

### 2.4. Data Analysis

Data from Section A of the questionnaire (Knowledge of AAI) were analysed descriptively (closed Yes/No question and open-ended questions about participants’ level of knowledge of and previous experience with AAI).

Data from Section B of the questionnaire (Implementation of AAI and their benefits) were analysed using frequency distributions (percentages of responses to multiple-choices questions).

Data from Section C of the questionnaire (Positive/negative aspects of AAI) were analysed qualitatively, using the principles of thematic analysis [20]. A primarily inductive approach was taken, focused on exploring topics and themes that emerged directly from the responses themselves [21]. The analysis process continued until saturation was reached; that is, until there were no new themes extracted from the data.

## 3. Results

A total of 44 respondents took part in the online survey (see Table 1 for participant characteristics). Most respondents were psychologists (43%) working in Lazio Region (62%). The limited number of Italian centres involved in AAI contributed to the small sample size.

### 3.1. Knowledge of AAI (Section A)

The majority of participants had knowledge of what AAI entails (72.7%), but without previous experience working with AAI (81.8%).

Of those who had worked with AAI in the past, one participant reported working in equine-assisted therapy, while others had worked with dogs within paediatric neurorehabilitation units. When asked to give a brief definition of AAI, several participants indicated a good knowledge of AAI; one participant described them as “interventions in which animals, having undergone specific training and together with trained personnel, work to reduce distress and improve quality of life in patients with various illnesses”. Others described AAI as: “Use of domestic animals (e.g., dog or horse) for therapeutic or rehabilitative ends”; “Interventions with animals intended to improve health and well-being of people”; and “Structured encounters between patient and animal aimed at promoting well-being of the patient”. In terms of what AAI can provide, some respondents wrote that these interventions can: “promote treatment adherence” and compliance; “make hospitalisation feel easier” and “reduce the use of pharmacological therapies”; “facilitate relationships with the external world and the expression of emotions”; “improve visual-motor coordination”; and provide “companionship during diagnostic-therapeutic procedures”. Also mentioned were the presence of a “multidisciplinary team” and use of AAI as a form of “co-therapy which works alongside traditional therapies”.

### 3.2. Implementation of AAI and Their Benefits (Section B)

Most participants selected “strongly agree” and “agree” to express their opinion of statements which described the ability of AAI to improve hospital environments (see Table 2). Similarly, most responses in the section “AAI as a resource for patients and families” tended to reflect agreement with statements describing how AAI may be a resource for young patients and families during hospitalisation. Responses to items in the section on hospital staff well-being were more varied, particularly Items 17 (“AAI would increase hospital staff responsibilities”), 18 (“AAI can help to prevent burnout in hospital staff”), and 19 (“AAI can promote bonding between hospital staff members (co-workers)”). Finally, responses to Items 21 and 22 indicate a weaker level of agreement compared to previous sections (in which most responses consisted primarily of “strongly agree” and “agree”). For example, in response to Item 21 (“The COVID-19 pandemic has affected my perception of AAI safety in paediatric hospitals”), 16 participants (36.4%) selected “disagree”, 11 (25%) chose “neither agree nor disagree”, 9 (20.5%) responded “strongly agree” and 8 (18.2%) chose “agree”. Responses to Item 22 (“Implementation of AAI is less feasible due to health and safety concerns that have arisen during the COVID-19 pandemic”) included “disagree” (17 respondents, 38.6%); “agree” (15 respondents, 34.1%); “neither agree nor disagree” (7 respondents, 15.9%); and “strongly disagree” (5 respondents, 11.4%).

### 3.3. Positive/Negative Aspects of AAI, Acceptance and AAI Implementation Challenges (Section C)

Results are shown describing the topics and themes that emerged from the responses, often including direct quotes from participants when illustrative.

#### 3.3.1. Positive Aspects of AAI

Qualitative analysis of responses to Item 23 (“What do you think the positive aspects of AAI are, if any?”) revealed a number of important themes, namely:

1. Positive impact on emotions/general well-being: Nineteen participants (43.2%) cited the ability of AAI to generate positive emotions and feelings (e.g., joy, tranquillity, love, serenity) and improve mood as a benefit of this form of intervention. They also described the capacity of AAI to enhance physical and especially mental well-being in not only patients, but also their families and healthcare personnel. Respondents stated how, by eliciting positive emotions, providing comfort and improving management of negative emotions such as fear and sadness, AAI can contribute to improved health outcomes. One participant wrote:

*“[AAI] can represent an important aid in extremely delicate situations that can occur in certain departments dedicated to the care of complex conditions. They serve as a distraction and also help to alleviate the stress experienced by the families of young patients.”*

(Participant 35, Nurse)

2. Compliance and impact on treatment: Another commonly noted benefit of AAI is their ability to improve compliance in patients and have a positive impact on treatment (10 participants, 22.7%). Some described how AAI can “increase motivation”, “make treatment more pleasant, acceptable, easier to face” and lead to “greater engagement with treatment”. One respondent cited: “overall improvement and reduced hospital stays for patients, and relief for family members” as a positive aspect of AAI. Another described “reductions in pharmacological treatments”. Other benefits mentioned were improved pain management and “greater satisfaction” with treatment on behalf of patients and their families.

3. Reduction in stress and distress: Ten participants (22.7%) felt that a positive aspect of AAI is their ability to reduce feelings of stress and distress in hospitalised children, their families, and healthcare operators, particularly during “surgical procedures and long recovery-times”. Some felt that AAI can also “reduce perception of suffering” in children and help make hospitalisation “less traumatic” for children.

4. Improved communication and relationships: Seven respondents (15.9%) cited improved communication and stronger relationships between patients, families, and healthcare personnel as a positive aspect of AAI. Multiple participants described how AAI can “increase relational availability” and have a positive impact on emotional relationships; one participant wrote:

*“Often AAI can help relationships to form even with children who are closed off emotionally.”*

(Participant 30, Psychologist)

Others identified the ability of AAI to promote openness through nonverbal means; one participant described AAI as allowing all parties to

*“(…) talk without using conventional language. These are democratic interventions, for everyone.”*

(Participant 4, Psychologist)

Similarly, another participant noted how AAI can engage and bring out spontaneity in children and their parents.

5. Distraction: Six participants (13.6%) cited distraction as a positive aspect of AAI, namely, its ability to distract children from the hospital environment in which they find themselves.

6. Positive impact on the environment: Five participants (11.4%) described how AAI can improve the hospital environment, creating a “positive climate” for children, families and hospital staff. One person wrote:

*“I would be in favour [of AAI] because I feel it is a way to alleviate patient stress, by bringing the outside world into an isolating context.”*

(Participant 40, Nurse)

Another respondent cited AAI’s ability to “facilitate decompression/unwinding in the hospital environment”; similarly, one participant described how AAI can “help hospitalised children to better accept the place in which they find themselves”.

#### 3.3.2. Negative Aspects of AAI

In response to Item 24 (“What do you think the negative aspects of AAI are, if any?”), 12 participants (27.3%) could not identify any negative aspects of AAI. Among the remaining responses, the main themes that arose were the following:

1. Organisational/logistical issues: This was the most described negative aspect of AAI, mentioned by 11 participants (25%). These difficulties included the fact that AAI “are not always available”; may be “difficult to manage”, carry out, and organise; and can be expensive. Several respondents also cited physical space as being a potential issue for the execution of AAI; one person wrote that this form of intervention “may not be suitable for the average Italian hospital structure”. Others identified difficulties in terms of managing time dedicated to these activities, as well as the handling of animals in appropriate spaces. Furthermore, one person replied:

*“The limited presence of [AAI] means that realistically they cannot be made available to all children.”*

(Participant 14, Psychologist)

Another mentioned the need for trained staff to take care of the animals as a negative aspect of this form of intervention.

2. Hygiene: Also commonly cited as a negative aspect of AAI were concerns around hygiene; 11 participants (25%) included this in their response, describing the difficulties with keeping hospital spaces clean and lack of hygiene safety.

3. Allergies, dislike, or fear of animals: Ten participants (22.7%) identified allergies in patients/family members and fear or dislike of animals as being potential negative aspects of AAI. More specifically, one person mentioned how allergies could lead to discrimination amongst children in terms of activities offered. Three participants (6.8%) also described a more general unwillingness or lack of acceptance of AAI by families and hospital staff as being potential negative aspects (“families could not be culturally prepared”; “mistrust on behalf of medical personnel”; “ignorance about animals”).

4. Impact on workload for hospital staff: Five participants (11.4%) felt that a negative aspect of AAI would be increased workload and responsibility for hospital staff. One respondent cited “possibility of eventual problems within departments (distraction, etc.)” while another felt that AAI could lead to

*“(…) slowing down of nursing duties, e.g., resulting in delayed withdrawal of a blood sample and subsequent delay in administration of anti-epileptic medication (which can then lead to epileptic seizure.”*

(Participant 17, Nurse)

#### 3.3.3. Willingness to Adopt AAI (Acceptance)

The results presented here are depicted in Table 3.

In response to Item 25 (“Would you be happy to welcome AAI into the hospital where you work? Why/why not? Please explain.”), six participants (13.6%) replied that AAI are already present in the hospital in which they work, and of these, two said that they would favour expansion of these activities. Thirty-three participants (75%) said “yes”; of these, four (9.1%) said “yes” but provided caveats, i.e., AAI should only take place in certain units; not in ‘cramped’ spaces; not during post-operative care; and only for “well-defined” cases. Additionally, one of these participants wrote that while they would be in favour of their hospital offering AAI, they are allergic to animals and acknowledged that this could be an issue both for patients and healthcare operators with the same problem. Among the rationales provided that were pro-AAI were “interesting experience with few collateral effects”; “benefit for patients and staff”; “new opportunity”; “indirectly promote cooperation between colleagues and minimise intensity of anxiety and fear in patients”; “animals stimulate empathy”; “facilitate adaptation to hospitalisation”; “for the benefits that an animal can bring like relaxation, socialisation, improvement of mood, prevention of depression”; “allowing better relationships to form with healthcare personnel”; “to improve working conditions”; “source of positive distraction for patients”; and “alleviate the patient’s stress by bringing the outside world into an isolated context”.

Of the five respondents (11.4%) who did not say “yes”, one replied “I don’t know”; another said that they wouldn’t welcome AAI into the hospital where they work because it is a “small hospital in the mountains with only one emergency room, medical unit, and dialysis machine”. Yet another wrote that offering AAI could not be managed in the hospital where they work both due to a lack of staff as well as other reasons; one respondent said that offering AAI would require more appropriate spaces, and another wrote they felt that the management or administration of AAI would be complicated.

#### 3.3.4. Challenges to AAI Implementation

In response to Item 26 (“What do you think are the challenges of implementing AAI in the hospital where you work, if any?”), nine participants (20.5%) responded “I don’t know” or “None”. The other challenges listed can be categorised as:

1. Organisational/logistical challenges: Twenty-five respondents (56.8%) cited practical difficulties with space (i.e., finding appropriate rooms for AAI to take place, limited space, etc.); expenses; time; and lack of adequately trained staff as potential challenges:

*“I fear that the costs [of AAI] may be higher than what our national health service is able to cope with.”*

(Participant 1, Doctor)

*“Hours dedicated to AAI should be increased because often they don’t manage to meet all requests and children are disappointed when they don’t get to meet with the animal.”*

(Participant 30, Psychologist)

Of these, six (13.6%) described specifically administrative and bureaucratic issues, i.e., challenges in obtaining necessary permits and relevant authorisation, insurance complications, etc. Other participants mentioned the challenges of “coordinating the different professional figures” in the multidisciplinary teams involved in AAI on a daily basis to discuss cases and to enact an “accurate evaluation of risks and subsequent interventions”. Integrating AAI in rehabilitation practices and other hospital activities was also identified as a potential challenge.

2. Resistance/Challenges to acceptance: Ten respondents (22.7%) felt that acceptance of AAI would be a challenge, with “people’s preconceived ideas and the bureaucracy of politicians” and “practical, management, and cultural” factors as a potential barrier to AAI. One person said a main challenge would be

*“(…) Making it such that AAI is always seen as an opportunity, and not as a burden.”*

(Participant 22, Psychologist)

While this was mentioned in the context of both patients/their families and medical staff, there was greater emphasis on resistance by medical staff. Respondents described the necessity of “improving behaviours and education of patients and healthcare personnel with regard to animals” and “inclusion of AAI on behalf of healthcare staff”; one participant identified “overcoming the suspicion/mistrust of doctors and healthcare management through exposure to scientific results [of AAI]” as a primary obstacle. Participants also mentioned the challenge of moving towards a more holistic/humanistic approach to care; one respondent described the difficulty of adopting AAI in a hospital climate in which

*“(…) traditional interventions are almost exclusively geared towards medicalisation.”*

(Participant 5, Doctor)

Similarly, another participant cited the challenge of

*“(…) making the relationship between healthcare users and patients easier and more focused on the humanisation of care.”*

(Participant 11, Psychologist)

3. Hygiene-related challenges: Five participants (11.4%) listed issues with hygiene as a potential challenge to AAI implementation. More specifically, one respondent said:

*“More frequent and thorough cleaning would be necessary [following AAI activities].”*

(Participant 20, Psychologist)

One respondent wrote of the prejudice associated with hygiene as a barrier. Another described the challenge of “overcoming restrictions still in force related to COVID”.

## 4. Discussion

### 4.1. Summary of Results

Results of this study are consistent with the existing literature on hospital personnel perspectives regarding AAI [7,8,9,10,11,12]. Most respondents expressed generally positive views and were in favour of AAI implementation in their respective hospitals. Only one participant held predominantly negative views; however, inconsistencies between this participant’s responses to multiple-choice and open-ended questions (the latter being primarily positive), suggest that this outlier may be attributed to error rather than due to genuinely negative opinions about AAI.

In response to questions regarding the hospital environment, the vast majority of participants “strongly agreed” or “agreed” with statements describing the ability of AAI to reduce stress, create a welcoming environment, provide a source of distraction, and improve treatment compliance. These findings are consistent with precious studies, such as Moody et al. (2002) [7], which showed that hospital staff had high expectations that AAI would distract children from their illness, relax them, and make the ward more cheerful. Similarly, most participants agreed that AAI serves as a resource for patients and families, e.g., by helping families manage feelings of stress during medical procedures, offering a non-judgmental and comforting presence during difficult experiences, and promoting overall well-being. This is in line with previous studies, which highlight AAI’s role in providing hospitalised children and their families with comfort and emotional support through therapy dogs [9,12].

In terms of impact on healthcare personnel, most participants agreed that AAI can have a positive impact on hospital staff morale, improve the workplace environment, and facilitate communication between children, their families, and hospital staff. However, responses varied on whether AAI increases workload. Similarly, most participants were uncertain (“neither agree nor disagree”) about whether AAI could prevent burnout among hospital staff and strengthen bonding among co-workers. This is consistent with mixed findings in the literature. For example, Rodriguez et al. (2022) [12] identified increased workload and responsibilities as potential downsides of AAI, particularly for facility dog handlers. It is worth noting that in the Italian context, hospital staff are not expected to manage therapy animals, unlike the handlers in Rodriguez et al.’s study. Conversely, Bibbo (2013) [18] reported no evidence of increased stress or workload due to AAI; the author concluded that AAI may be introduced into facilities without adding significant burden to staff members.

In terms of AAI implementation feasibility during the COVID-19 pandemic, most participants felt that the pandemic had not affected their perception of AAI safety in paediatric hospitals, though several participants remained uncertain (“neither agree nor disagree”). This may reflect the stringent mitigation measures adopted across Italian hospitals during the pandemic, as Italy was the first and most affected European country, and its government the first Western government to implement strict mitigation measures early on to control the outbreak [22].

The most frequently identified benefits of AAI included its positive impact on emotions, general well-being, treatment compliance, stress reduction, improved communication and relationships, distraction, and its ability to create a more welcoming environment. These benefits are well-documented in the literature within the context of paediatric hospital settings [12,23]. Negative aspects of AAI that were identified by respondents included organisational/logistical issues (including expenses, space, and time management), concerns about hygiene, allergies, dislike or fear of animals, and impact on staff workload. These concerns are consistent with previous studies [9,12,24]. In Italy, organisational and financial challenges may be particularly pronounced due to the multidisciplinary team requirements and extensive training outlined in the national guidelines, coupled with ongoing funding issues in the Italian National Health Service [25].

Organisational and logistical challenges were indeed the most perceived barriers to AAI implementation in our study; these included limited spaces, budget constraints, time management, lack of adequately trained staff, administrative/bureaucratic issues, and the integration of AAI with existing hospital activities. These concerns mirror widespread heterogeneity in the literature regarding AAI implementation, highlighting the need for standardised operational and research protocols [26].

Cultural acceptance emerged as the second most reported challenge, with participants highlighting scepticism among patients, their families, and hospital staff, primarily doctors and administrators. This scepticism may stem from an underestimation of the therapeutic value of the animal–human bond, and the clinical utility of animals as an adjunct to therapy [27].

Lastly, hygiene-related concerns, including those associated with COVID-19 restrictions, were also reported. While previous research suggests that AAI can be safely implemented with minimal risk of virus transmission between humans and animals [16,28], the mixed responses in this survey highlight the need for greater reassurance regarding adequate hygiene measures when implementing AAI.

Addressing staff concerns requires institutional-level efforts to enhance knowledge about AAI, ensure compliance with national guidelines set by the Italian Ministry of Health, uphold safety standards for human participants, and protect the welfare of the animals involved.

### 4.2. Strengths and Limitations

To the authors’ knowledge, this is the first study to explore paediatric hospital staff’s attitudes towards AAI across multiple regions of Italy, beyond the context of a single pilot program (Caprilli & Messeri, 2006 [9]). Negatively framed items were included to limit response bias.

However, our findings should be interpreted considering the study’s limitations. Given that AAI remains relatively uncommon in Italian paediatric hospitals, the pool of eligible respondents was limited, which may have influenced the generalisability of the findings. The relatively small sample size, particularly the limited representation from southern Italy, may reflect disparities in paediatric healthcare services across regions [29]. Further studies with larger and more diverse samples should be conducted to confirm and expand upon our results.

Data collection—which did not include sex and age for privacy reasons—limited the ability to explore how factors like pet ownership or hospital roles might influence staff’s attitudes toward AAI. Finally, the use of the convenience sampling method—while practical for reaching a diverse group in a short timeframe—may limit the generalisability of findings due to potential sampling bias. A more structured qualitative approach could be valuable for future research, such as in-depth interviews or focus groups, to explore this topic more deeply. Despite these limitations, this study provides valuable preliminary insights and identifies areas for further research.

## 5. Conclusions

As highlighted by this and previous studies, most hospital staff hold positive views toward AAI. Future research could build on these findings by exploring these attitudes within the context of specific programs, allowing for the monitoring and evaluation of successful implementation on a case-by-case basis. However, concerns regarding organisational and logistical challenges, hygiene, allergies, fear of animals, and the potential impact on work responsibilities must be carefully addressed, particularly at the institutional level. By addressing these challenges, the full benefits of AAI can be realised for children, families, and healthcare personnel alike.

## Figures and Tables

**Table 1:** Respondent characteristics.

Profession		N (%)
	Psychologist	19 (43%)
	Nurse	12 (28%)
	Doctor	10 (23%)
	Administrator	1 (2%)
	Healthcare assistant (OSS)	1 (2%)
	Other	1 (2%)
Region of Italy		
	Lazio	27 (62%)
	Toscana	6 (14%)
	Piemonte	4 (9%)
	Calabria	2 (5%)
	Lombardia	1 (2%)
	Liguria	1 (2%)
	Marche	1 (2%)
	Puglia	1 (2%)
	Emilia Romagna	1 (2%)

**Table 2:** Participants’ perspectives on the benefits of AAI implementation.

Section	Item	Strongly Disagree	Disagree	Neither Agree nor Disagree	Agree	Strongly Agree
AAI and the hospital environment						
	5. AAI can make the hospital environment less stressful for children and their families.	1 (2.3%)	0	1 (2.3%)	14 (31.8%)	28 (63.6%)
	6. AAI can help to create a more welcoming environment for children and their families.	1 (2.3%)	0	1 (2.3%)	14 (31.8%)	28 (63.6%)
	7. AAI can make the hospital environment more acceptable to children and their families by providing a source of distraction.	1 (2.3%)	0	1 (2.3%)	16 (36.4%)	26 (59.1%)
	8. AAI can help patients be more likely to accept treatment and/or medical procedures.	1 (2.3%)	0	8 (18.2%)	11 (25%)	24 (54.5%)
AAI as a resource for patients and families						
	9. The presence of AAI in the hospital can help families to manage feelings of stress during their child’s medical procedures.	1 (2.3%)	0	7 (15.9%)	15 (34.1%)	21 (47.7%)
	10. AAI can help families feel more at ease by offering a comforting and non-judgmental presence.	1 (2.3%)	0	6 (13.6%)	18 (40.9%)	19 (43.2%)
	11. I think that children and their families would be pleased to have AAI as a hospital service.	1 (2.3%)	0	2 (4.5%)	17 (38.6%)	24 (54.5%)
	12. AAI can help promote feelings of well-being in hospitalised children.	1 (2.3%)	0	0	15 (34.1%)	28 (63.6%)
	13. AAI can have a calming effect on hospitalised children.	1 (2.3%)	0	0	14 (31.8%)	29 (65.9%)
	14. AAI can provide children and families with comfort during difficult experiences (e.g., grieving or being told a diagnosis).	1 (2.3%)	0	6 (13.6%)	12 (27.3%)	25 (56.8%)
AAI and hospital staff well-being						
	15. AAI can have a positive impact on hospital staff morale.	1 (2.3%)	0	8 (18.2%)	17 (38.6%)	18 (40.9%)
	16. AAI can improve the work environment for hospital staff.	1 (2.3%)	0	11 (25%)	14 (31.8%)	18 (40.9%)
	17. AAI would increase hospital staff workload and responsibilities.	4 (9.1%)	9 (20.5%)	14 (31.8%)	14 (31.8%)	0
	18. AAI can help to prevent burnout in hospital staff.	1 (2.3%)	5 (11.4%)	18 (40.9%)	12 (27.3%)	8 (18.2%)
	19. AAI can promote bonding between hospital staff members (co-workers).	1 (2.3%)	1 (2.3%)	19 (43.2%)	13 (29.5%)	10 (22.7%)
	20. AAI can help to facilitate communication between children and their families, and hospital staff.	0	0	8 (18.2%)	16 (36.4%)	20 (45.5%)
Perceptions of AAI implementation feasibility						
	21. The COVID-19 pandemic has affected my perception of AAI safety in paediatric hospitals.	9 (20.5%)	16 (36.4%)	11 (25%)	8 (18.2%)	0
	22. Implementation of AAI is less feasible due to health and safety concerns that have arisen during the COVID-19 pandemic.	5 (11.4%)	17 (38.6%)	7 (15.9%)	15 (34.1%)	0

**Table 3:** Participants’ willingness to adopt AAI.

“Would You Be Happy to Welcome AAI Into the Hospital Where You Work? Why/Why Not? Please Explain.”	Number of Respondants (tot = 44)	Percentage (%)
AAI already present	6	13.6%
→ Favor to expansion	2	4.6%
Yes	33	75%
→ Yes—with caveats	4	9.1%
“Only in certain units”		
“Not in cramped spaces”		
“Not during post-operative care”		
“Only for well-defined cases”		
No	5	11.4%
“Not sure”		
“Small hospital limitations”		
“Lack of staff & other issues”		
“Requires more appropriate spaces”		
“Complicated management”		

## Data Availability

Data presented in this study are available on request from the corresponding author. The data are not publicly available due to restrictions, e.g., privacy or ethical.

## Abbreviation

AAI	Animal-assisted intervention
